# The Effects of Divalent Cation-Chelated Prion Fibrils on the Immune Response of EOC 13.31 Microglia Cells

**DOI:** 10.3390/cells9102285

**Published:** 2020-10-13

**Authors:** Huan-I Jen, Zih-You Lin, Jin-Xun Guo, Cheng-I Lee

**Affiliations:** 1 Department of Biomedical Sciences, National Chung Cheng University, 168 University Road, Min-Hsiung Chia-Yi 62102, Taiwan; e3488176@gmail.com (H.-I.J.); qaz0970603641@gmail.com (Z.-Y.L.); syun840223@gmail.com (J.-X.G.); 2 Center for Nano Bio-Detections, National Chung Cheng University, Min-Hsiung Chia-Yi 62102, Taiwan; 3 Center for Innovative Research on Aging Society (CIRAS), National Chung Cheng University, Min-Hsiung Chia-Yi 62102, Taiwan

**Keywords:** prion, microglia, inflammation, NLRP3 inflammasome

## Abstract

Transmissible spongiform encephalopathies (TSEs) are epidemic neurodegenerative diseases caused by prion proteins; in particular, they are induced by misfolded prion proteins (PrP^Sc^). PrP^Sc^ tend to aggregate into insoluble amyloid prion fibrils (fPrP^WT^), resulting in apoptosis of neuron cells and sequential neurodegeneration. Previous studies indicate that microglia cells play an important role in the innate immune system, and that these cells have good neuroprotection and delay the onset of TSEs. However, microglia can be a double-sided blade. For example, both Cu^2+^ and Mn^2+^ can induce microglia activation and secrete many inflammatory cytokines that are fatal to neuron cells. Unfortunately, PrP have cation binding sites at the N-terminus. When PrP^Sc^ accumulate during microglial phagocytosis, microglia may change the phenotype to secrete pro-inflammation cytokines, which increases the severity of the disease. Some studies have revealed an increase in the concentration of Mn^2+^ in the brains of patients. In this study, we treated microglia with fPrP^WT^ and cations and determined IκBα and IL-1β expression by Western blotting and quantitative polymerase chain reaction. The results showed that Mn–fPrP^WT^ decreased IκBα levels and dramatically increased IL-1β mRNA expression. In addition, competing binding between Cu^2+^ and Mn^2+^ can decrease the effect of Mn–fPrP^WT^ on IκBα and IL-1β. The effects of divalent cations and fPrP^WT^ in microglia inflammation are also discussed.

## 1. Introduction

Transmissible spongiform encephalopathies (TSEs), also called prion diseases, are neurodegenerative diseases. Mammals can become infected with TSEs through inherent, transmitted, and sporadic conditions [1]. In humans, the population that contracts TSEs through sporadic prion conditions is higher (more than 85% of patients) than the transmitted and inherent populations [2,3,4]. Transmitted prion diseases, such as the variant Creutzfeldt–Jakob disease (vCJD), have drawn more attention and are mainly ascribed to infection through blood or viscera [5,6]. Scrapie prion proteins (PrP^Sc^), abnormal isoforms of cellular prion proteins (PrP^C^), are pathogens of prion diseases. Structurally, PrP^C^ are rich in α-helices, whereas PrP^Sc^ are rich in β-sheets. Once PrP^Sc^ present in the central nervous system (CNS), PrP^Sc^ can serve as a template for PrP^C^ to process a structural conversion from an α-helix-rich structure to a β-sheet-rich structure [7,8]. The accumulation of misfolded PrP^Sc^ can induce brain inflammation, neuron degeneration, and plaque aggregation [9,10]. Patients carrying prion diseases rapidly develop progressive dementia with behavioral abnormalities, ataxia, and myoclonus [9].

Prion proteins contain a flexible N-terminal domain and a structured C-terminal domain. In the N-terminal domain, there are five contiguous copies of octapeptides (P(H/Q)GG(G/S/T)WGQ) called octapeptide repeats (ORs) [11]. Among these octapeptides, four of them contain histidine residues to bind divalent cations such as copper and manganese ions. The repetition of ORs affects the age of onset in patients [12]. High concentrations of divalent cations in the CNS can accelerate fibril conversion [13] and can, therefore, enhance the toxicity in neuron cells [14]. According to a blood investigation, patients with neurodegenerative diseases have a high concentration of heavy metal ions [13]. Sporadic Creutzfeldt–Jakob disease (sCJD) patients have higher concentrations of copper and manganese ions, while patients with Alzheimer’s disease have higher concentrations of copper ions, and patients with Parkinson’s disease have higher concentrations of iron and zinc ions [15]. However, an analysis of TSE-infected mouse/human brain homogenates using X-ray photoelectron emission microscopy indicated reduced levels of copper and increased levels of manganese [16,17]. Furthermore, Cu^2+^-binding in the ORs of PrP^C^ can protect cells against oxidative stress. This protection is lost when PrP^C^ are converted into PrP^Sc^ [18]. Therefore, the divalent cations in the CNS are important and complicated in neurodegenerative diseases.

Microglia are the primary immune cells in the CNS. Microglia are responsible for maintaining the brain’s homeostasis and play an important role in prion diseases. Microglia occupy approximately 10% of healthy brain and spinal cord cells [19], and constantly patrol brain parenchyma and sensitively respond to environmental changes in the brain [20]. Upon abnormal changes in the environment, microglia in the homeostasis state (M0) are activated into a pro-inflammatory state (M1) or an anti-inflammatory state (M2). The physiological response in this microglia activation includes killing abnormal cells, pathogen clearance, and cytokine secretion. In neurodegenerative diseases, microglia cells are turned into activated states (i.e., M1 and M2) [19]. The M1 state secretes inflammatory cytokines, such as IL-1β and tumor necrosis factor-α (TNF-α), to cause apoptosis of neuron cells [21]. The M2 state secretes anti-inflammatory cytokines, such as TGF-β and IL-10, to protect cells and to inhibit the production of pro-inflammation cytokines. The switch between the M0 and M1/M2 states depends on the factors in milieu and stimulated microglia possess states of classical activation, alternative activation, and acquired deactivation [22,23]. Classical activation can induce pro-inflammatory cytokines, such as TNF-α, IL-1β, and NO [24,25]. Alternative activation is associated with the M2 state and is only activated by IL-4 and IL-13 [23]. Acquired deactivation can alleviate acute inflammation and can be induced by uptake of apoptosis cells, i.e., IL-10 and transforming growth factor-β (TGF-β) [22,26].

**Figure 1:**
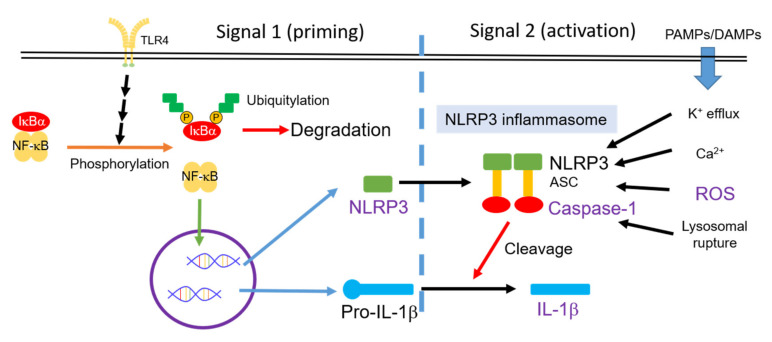
Activity of the NLRP3 inflammasome is controlled by two signal pathways: Priming and activation in sequence [29]. PAMPs, pathogen-associated molecular pattern molecules; DAMPs, damage-associated molecular patterns; ROS, reactive oxygen species; NLRP3, nucleotide-binding and oligomerization domain-like receptor family pyrin domain-containing 3.

Activation of the nucleotide-binding and oligomerization domain-like receptor family pyrin domain-containing 3 (NLRP3) inflammasome indicates chronic inflammation in a variety of inflammatory cells [27]. As shown in Figure 1, the main components of the NLRP3 inflammasome are NLRP3 protein, apoptosis-associated speck-like protein containing a CARD (ASC), and pro-caspase-1 [28]. The activity of the NLRP3 inflammasome requires upstream signals of priming and activation in sequence [29]. Priming, the first signal, can be triggered by microbial molecules (e.g., lipopolysaccharide (LPS)) or by endogenous cytokines (e.g., TNF-α). This priming signal can increase the activity of NF-κB, resulting in the high expression of NLRP3 and pro-IL-1β. Activation, the second signal, can be triggered by multiple damage-associated molecular patterns (DAMPs) and pathogen-associated molecular pattern molecules (PAMPs). Moreover, K^+^-efflux, Ca^2+^, reactive oxygen species (ROS), and lysosomal ruptures participate in the mechanism of the NLRP3 inflammasome activation [29]. Pro-caspase-1 is recruited to the NLRP3 inflammasome and processed into caspase-1 via autocatalysis. Caspase-1 can cleave pro-inflammatory cytokine such as pro-IL-1β and pro-IL18 [28]. In addition, Mn^2+^ induces activation of the NLRP3 inflammasome, resulting in pro-caspase-1 cleavage [30]. These sequential signals can promote pro-IL-1β cleavage, can secrete more IL-1β, and can induce inflammation in neurons.

As aforementioned, recent studies have revealed the correlation between neurodegenerative diseases and microglia. However, the effect of metal-chelated prion fibrils in microglia remains ambiguous. In this research, we prepared prion proteins with or without ORs and their fibrils. We treated microglia cells with fibrils chelated with copper and/or manganese ions to study inflammation-associated mRNA. The effect of Cu^2+^- and Mn^2+^-chelated prion fibrils were investigated.

## 2. Materials and Methods

### 2.1. Prion Purification

The wild-type mouse prion plasmid pET101 with insertion of PrP23–231 (PrP^WT^) and PrP^ΔOct^ genes [31] were transformed to BL21 *Escherichia coli* (*E. coli*.) individually. After protein overexpression induced by isopropyl β-D-1-thiogalactopyranoside, *E. coli* was disrupted by French press or lysozyme treatment. Inclusion bodies were collected by centrifugation and then dissolved in urea- and glutathione-containing buffer at pH 8.5 according to a previously described procedure [32]. For PrP23–231, the soluble inclusion bodies were mixed with nickel-chelating Sepharose for further experimentation with immobilized metal affinity chromatography. Prion proteins were eluted with urea-containing buffer at pH 4. For PrP^ΔOct^, the protein was purified by sulphopropyl (SP) Sepharose cation exchange chromatography. Fractions containing prion proteins were refolded in glutathion disulfide overnight. The refolded prion proteins were further purified with a C4 reversed-phase high-performance liquid chromatography (HPLC) column under an elution of acetonitrile/water gradient. After lyophilization, the purified protein powders were stored at −80 °C. The purity of the prion was confirmed by SDS-PAGE.

### 2.2. Fibril Conversion

Fibril conversion starting from 0.25 μg/μL PrP^WT^ or PrP^ΔOct^ was performed in buffer solution containing 50 mM of 2-(N-morpholino) ethanesulfonic acid (MES, pH 6.5), 2 M of GdnHCl, and 2.5 ng/μL of PrP^WT^ or PrP^ΔOct^ fibril seeds at 37 °C under shaking as described previously [33]. The progress of fibril formation was monitored by fluorescence intensity of thioflavin T (ThT) for its binding to the cross-β-sheets in the fibrils. The fibrils converted from PrP^WT^ and PrP^ΔOct^ are denoted as fPrP^WT^ or fPrP^ΔOct^, respectively. The morphology of fPrP^WT^ was examined by transmission electron microscopy (TEM) according to a previously described procedure [33]. The fibril samples were dialyzed against water and then mixed with CuCl_2_ or MnCl_2_ for further experiments. The binding of copper ions to fPrP^WT^ was examined using a Copper Assay Kit (Sigma-Aldrich, St. Louis, MO, USA).

### 2.3. Cell Culture

The immortalized mouse brain EOC 13.31 microglia cells (ATCC CRL-2468) were purchased from the Bioresources Collection and Research Center (BCRC, Hsinchu, Taiwan). The condition medium containing CSF-1 for EOC 13.31 culture was collected from mouse bone marrow cell LADMAC (ATCC CRL-2420) after five to seven days of culture. The culture medium of EOC 13.31 cells contained Dulbecco’s modified Eagle medium (DMEM) supplemented with 4 mM of L-glutamine, 1.5 g/L of sodium bicarbonate, and 4.5 g/L of glucose at pH 7.4. After filtering the DMEM with a 0.22 μM filter cup, the condition medium and fetal bovine serum (FBS) were added to the DMEM to reach final concentrations at 20% and 10%, respectively. The LADMAC was cultured in Eagle’s Minimum Essential Medium (EMEM) supplemented with 1.5 g/L of sodium bicarbonate, 0.1 mM of non-essential amino acids, 1.0 mM of sodium pyruvate, and 10% FBS.

### 2.4. Detection of Cell Viability and Cellular Reactive Oxygen Species

EOC 13.31 cells were cultured in 96-well plates and incubated overnight. In the cell viability assays, EOC 13.31 cells were treated with fPrP and cations for 24 h, and then 10 μL Cell Counting Kit 8 (CCK8) reagent was added for 40 min. Cell viability was determined based on the decrease of OD450 recorded by a plate reader (Thermo Fisher Scientific, Waltham, MA, USA). To detect the cellular ROS in the EOC 13.31 cells, the microglia cells were cultured in 96-well black plates and incubated overnight. After treatment of fPrP and cations for 4 h, 50 μM of 2′,7′-dichlorofluorescin diacetate (DCFDA) was added and incubated for 40 min. Fluorescence data were recorded via a fluorescence plate reader (BMG LABTECH, Offenburger, Germany).

### 2.5. Western Blot

A radioimmunoprecipitation assay (RIPA) lysis buffer with protease and a phosphatase inhibitor was used to collect cell lysate after washing with PBS. The cells were lysed with RIPA lysis buffer for 15 min on ice and then collected by cell scrapers. The collected supernatant was centrifuged at 12,000× *g* for 10 min and stored at −20 °C. The cell lysate was diluted by 2× sample buffer and then heated at 95 °C for 5 min. Gel electrophoresis was performed with 12.5% SDS-PAGE. Afterward, wet polyvinylidene fluoride (PVDF) membranes were soaked in methanol for at least 15 min. Transfer buffer containing 0.37 M of glycine, 46.2 mM of Tris-base, and 20% methanol (*v/v*) was used to transfer proteins onto the PVDF membrane under 400 mA for 2 h at 4 °C. The membranes were blocked with 5% skimmed milk in phosphate buffered saline with Tween^®^ 20 (PBST) for 1 h and then washed with PBST. Afterward, primary antibody diluted 1000 fold with 5% skimmed milk in PBST was used to interact with the membrane under shaking at room temperature for 1 h. Four primary antibodies were used: ab9722, ab263899, and ab32518 from Abcam for IL-1β, NLRP3, and IκBα, respectively, and A5441 from Sigma for β-actin. After washing with PBST, secondary antibodies (A9044 and A0545 from Sigma for anti-mouse IgG and anti-rabbit IgG, respectively) diluted 100,000 fold with 5% skimmed milk in PBST were used for further incubation for 1 h at room temperature. The Western blot membranes were imaged by a luminescence imaging system (TopBio, Czechia) and analyzed using Image J software (National Institutes of Health, MD, USA).

### 2.6. Extraction of mRNA

Cells were dissociated from culture dishes by 0.05% trypsin. Subsequently, the medium was removed by centrifugation. The cell pellets were washed twice by PBS. Cells were lysed by incubation with 1 mL of TRIzol^®^ reagent for 5–10 min. Afterward, 200 μL chloroform was added for mRNA extraction. The extracted mRNA was precipitated by 50% isopropanol. The mRNA precipitate was then washed with 75% ethanol (diluted with diethyl pyrocarbonate-treated water) and then spun down. Finally, the mRNA was air-dried and stored at −20 °C until further measurements.

### 2.7. Quantitative PCR

Total mRNA was treated with DNase (Invitrogen, Carlsbad, CA, USA) to remove residual DNA, then converted to cDNA with Moloney Murine Leukemia Virus Reverse Transcriptase (M-MLV RT, Invitrogen, Carlsbad, CA, USA) for 50 min at 37 °C. The reaction was terminated by heating polymerase at 70 °C for 10 min. The Power SYBR Green PCR Master Mix Kit (Applied Biosystems, Foster City, CA, USA) was used to run quantitative PCR (qPCR). Each well contained 20 ng of cDNA, 10 μL of SYBR green mix, 10 μM of forward primer, and 10 μM of reverse primer. In addition, diethyl pyrocarbonate (DEPC)-treated water was added to the total sample volume of 20 μL. The primers used for the qPCR were IL-1β forward, 5′-TCC TCT CCA GCC AAG CTT CC-3′, reverse, 5′-GGT TTG GAA GCA GCC CTT CAT-3′; GAPDH forward, 5′-AGG TCG GTG TGA ACG GAT TTG-3′, reverse, 5′-TGT AGA CCA TGT AGT TGA GGT CA-3′. In the qPCR, the temperatures were set at 60 °C and 72 °C for annealing and elongation, respectively. The relative amount of IL-1β mRNA was determined using the comparative Ct (ΔΔCt) method by normalizing the target mRNA Ct values to GAPDH. All reported data were obtained from triplicate measurements.

### 2.8. Statistical Analysis

All reported data were collected from three independent experiments, and values were analyzed by the Student’s *t*-test using GraphPad 5.0 software. The images of Western blotting were analyzed by Image J software. Statistical significance is shown using asterisks (ns: *p* > 0.05; *: *p* < 0.05; **: *p* < 0.005; ***: *p* < 0.0005).

## 3. Results

### 3.1. The Effect of Divalent Cations and Fprpwt on the Production of Cellular Ros in Eoc 13.31 Microglia Cells

**Figure 2:**
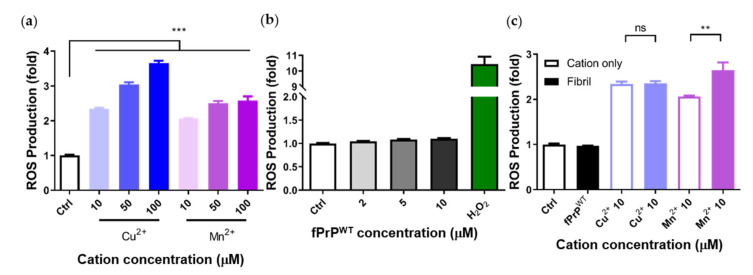
Determination of cellular ROS induced by fPrP^WT^ or by cations based on 2′,7′-dichlorofluorescin diacetate (DCFDA) fluorescence. (**a**) EOC 13.31 microglia treated with copper and manganese ions. (**b**) EOC 13.31 microglia treated with fPrP^WT^ and 100 μM of H_2_O_2_. (**c**) EOC 13.31 microglia treated with pre-mixed 2 μM of fPrP^WT^ and 10 μM of copper or manganese ions. Statistical significance is shown using asterisks (ns: *p* > 0.05; **: *p* < 0.005; ***: *p* < 0.0005).

The kinetics of fPrP^WT^ formation is shown in the time course of ThT fluorescence, and the morphology of fPrP^WT^ is shown in a TEM image (Figure S1). To examine the effect of divalent cations and fPrP^WT^ on the cellular ROS production in microglia, we determined the production of cellular ROS based on the fluorescence of DCFDA in EOC 13.31 cells. As shown in Figure 2a, cellular ROS increased when treated with copper and manganese ions in a dose-dependent manner. In contrast, cellular ROS production was not related to the dose of fPrP^WT^ (Figure 2b). These results indicate that copper or manganese ions, rather than fPrP^WT^, can induce ROS in microglia cells. In Figure 2c, one can see that fPrP^WT^ did not increase ROS levels with the addition of copper, but it slightly increased the levels of ROS with the addition of manganese. It is known that PrP^C^ can decrease manganese uptake and ROS level in cells [34]. Therefore, we propose that the PrP^C^ to PrP^Sc^ conversion caused the loss of PrP^C^ function. As a result, the cellular manganese and ROS levels were enhanced. In addition to the ROS assay, the viability of the cells after treatment with fPrP^WT^ and cations for 24 h was tested. As shown in Figure S2, cation-bound fPrP^WT^ caused weak cell death. The cells were in good condition for ROS determination.

### 3.2. The Effect of Divalent Cations on the Expression of nlrp3 and IκBα in Fprpwt-Treated Microglia Cells

**Figure 3:**
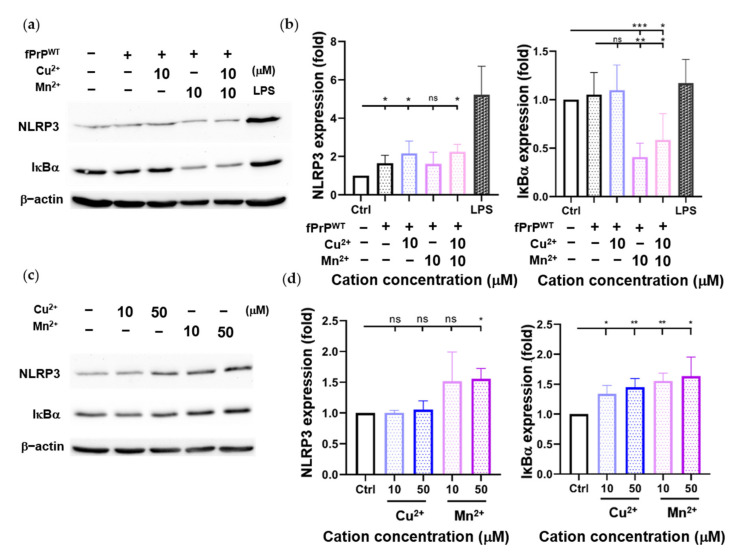
Determination of NLRP3 and IκBα protein levels in EOC 13.31 microglia cell lysate by Western blotting. (**a**) A Western blot image of NLRP3 and IκBα proteins collected after treating EOC 13.31 cells with 2 μM of fPrP^WT^ and/or 10 μM of Cu^2+^ and/or Mn^2+^ for 24 h. (**b**) Quantification of NLRP3 and IκBα protein levels shown in (**a**). (**c**) A Western blot image of NLRP3 and IκBα proteins collected after treating EOC 13.31 cells with copper and manganese ions for 24 h. (**d**) Quantification of NLRP3 and IκBα protein levels shown in (**c**). Statistical significance is shown using asterisks (ns: *p* > 0.05; *: *p* < 0.05; **: *p* < 0.005; ***: *p* < 0.0005).

Cellular ROS cause mitochondrial damage and stimulate NLRP3 inflammasome activation [30,35]. Although fPrP^WT^ cannot induce cellular ROS in EOC 13.31 cells, it can slightly increase the level of NLRP3 (Figure 3b). According to Figure 1, fPrP^WT^ might activate NF-κB in microglia. IκBα is an inhibitor of NF-κB. When IκBα is phosphorylated and degraded, the active NF-κB is released and enters the nucleus to promote target gene expression such as NLRP3, IL-1β, or IL-18. Treatment with Mn–fPrP^WT^ can severely decrease IκBα protein levels (Figure 3a,b). This indicates that NF-κB activity is increased in Mn–fPrP^WT^ treatment. In contrast, Cu^2+^-chelated fPrP^WT^ (Cu–fPrP^WT^) does not activate NF-κB in microglia, and treatment with Cu^2+^ or Mn^2+^ does not decrease IκBα protein levels (Figure 3c,d). Therefore, the increase of NF-κB activity is ascribed to Mn–fPrP^WT^ rather than free Mn^2+^. There is no difference between the control and 10 μM of Cu^2+^ or Mn^2+^ treatment in NLRP3 levels. Notably, Mn–fPrP^WT^ increases NF-κB activity, but it does not affect NLRP3 expression.

### 3.3. The Effect of Divalent Cations on the Expression of IL-1β Mrna in Fprpwt-Treated Microglia Cells

**Figure 4:**
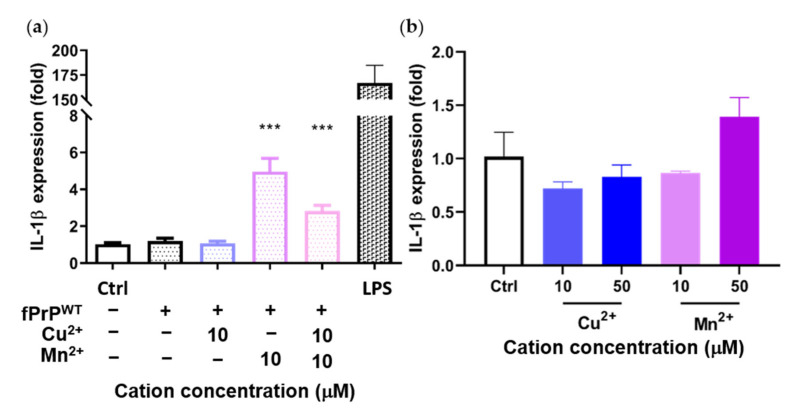
Expression of IL-1β mRNA in EOC 13.31 microglia cells determined by qPCR. The cells were treated with (**a**) fPrP^WT^ chelated with 10 μM of copper and/or manganese ions and (**b**) 10 μM and 50 μM of copper or manganese ions without fPrP^WT^ for 24 h. Statistical significance is shown using asterisks (***: *p* < 0.0005).

Previous results showed that Mn–fPrP^WT^ increases NF-κB activity. Therefore, we tested the mRNA level of IL-1β, another NF-κB downstream protein, by qPCR. Mn–fPrP^WT^ was able to upregulate the mRNA levels of IL-1β, but copper chelation had no such effect on the microglia cells (Figure 4a). Notably, IL-1β mRNA levels were not affected by treatment with fPrP^WT^ or Cu^2+^ (Figure 4a,b). This result shows that only Mn–fPrP^WT^ can promote the priming signal in the NLRP3 inflammasome by inducing NF-κB activity and the sequential upregulation of IL-1β mRNA in microglia.

### 3.4. The Effect of Octapeptide Repeat of Prions on the Expression of Inflammatory Cytokines in Microglia Cells

**Figure 5:**
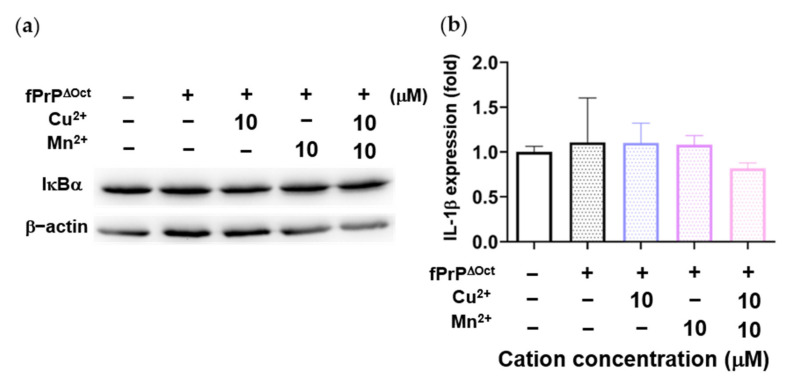
IκBα levels and IL-1β mRNA expression in EOC 13.31 cells treated with fibrils converted from octapeptide-deleted prions (fPrP^ΔOct^) with copper and manganese ions. (**a**) IκBα protein levels in 2 μM of fPrP^ΔOct^ and 10 μM of cation-treated microglia. (**b**) IL-1β mRNA expression in 2 μM of fPrP^ΔOct^ and 10 μM of cation-treated microglia.

As Figure 3 and Figure 4 indicate, Mn–fPrP^WT^ rather than fPrP^WT^ was able to activate NF-κB, the metal binding sites of prions critical in microglia inflammation. The binding of copper ions in fPrP^WT^ is shown in Figure S3. As divalent cations bound to octapeptide repeat regions of prion proteins, we used fibrils converted from octapeptide-deleted prions (fPrP^ΔOct^) to perform the above experiments. Mn–fPrP^ΔOct^ treatment did not change the expression of IκBα (Figure 5a) and IL-1β (Figure 5b). This result indicates that Mn–fPrP^ΔOct^ does not activate NF-κB as Mn–fPrP^WT^ does. Conclusively, the binding of manganese ions on prion fibrils is critical for inducing the priming signal in the NLRP3 inflammasome.

## 4. Discussion

Prion proteins are a well-known Cu^2+^-binding proteins with four strong binding sites [36]. Prion proteins can bind Mn^2+^ with moderate affinity and are considered a manganese-binding protein [37]. Manganese ions have been found to be neurotoxic to the human brain and cause cognitive dysfunction and motor impairment resembling Parkinson’s disease [38]. The concentration of manganese ions in the brain of prion disease patients is higher than that in healthy brains [16], especially in accumulated plaques [15]. Other than copper and manganese ions, zinc and iron ions are also essential metal ions in humans. In terms of iron, PrP^C^ are considered to modulate iron homeostasis with the Zrt, Irt-like protein (ZIP) family [39], but the role of iron in the inflammation associated with prion disease is ambiguous. In regards to zinc, there are many studies focusing on zinc as an antagonist with copper [40], and PrP^C^ can induce the uptake of zinc in neurons [41]. In addition, an increase in zinc levels decreases the activity of the NLRP3 inflammasome [42,43]. Therefore, a zinc replacement in PrP^Sc^ can potentially inhibit inflammation in microglia.

In previous research, fPrP^WT^ was shown to activate the NLRP3 inflammasome and IL-1β cleavage, but was not able to induce activation of NF-κB [44]. Therein, to activate the priming signal, LPS was added prior to the treatment of fPrP^WT^. In our results, a low concentration of Mn–fPrP^WT^ severely increased IL-1β mRNA levels by activating NF-κB in microglia cells. Conclusively, Mn–fPrP^WT^ can initiate the priming signal but fPrP^WT^ can initiate the activation signal in the NLRP3 inflammasome. Taking Mn–fPrP^WT^ and fPrP^WT^ in sequence can produce IL-1β in microglia.

Our results reveal that manganese ions enable fPrP^WT^ to activate NF-κB through their binding to ORs. This effect may come from microglia phagocyte PrP^Sc^ that uptake manganese and increase the concentration in cells. In addition, octapeptide repeat numbers can affect the age of onset of patients [12]. Enlargement of the ORs copy number may increase the amount of manganese on ORs and then increase the sensitivity of manganese in microglia.

We also observed that NF-κB activity and IL-1β mRNA levels were slightly lower when both Mn^2+^ and Cu^2+^ were added into the fPrP^WT^. As reported previously, the binding affinity of Cu^2+^ is higher than that of Mn^2+^ [37]. Cu^2+^ may compete with Mn^2+^ in ORs; therefore, the effect of Mn–fPrP^WT^ on NF-κB activation was weakened by the Cu^2+^ addition. Overall, the binding of manganese ions on prion fibrils is critical for inducing the priming signal in the NLRP3 inflammasome.

PrP^Sc^ are notorious for increasing IL-1β and for inducing neurotoxicity [45]. Herein, we propose that PrP^Sc^ have a positive effect as a double-edged sword in microglia activation. This activation can decrease prion infectivity and progression by phagocytosis [46]. Therefore, it is not always an advantage to indiscriminately inhibit the activation of microglia.

## Supplementary Materials

The following are available online at https://www.mdpi.com/2073-4409/9/10/2285/s1, Figure S1: The kinetics and TEM image of fPrP. Figure S2: The cell viability of EOC 13.31 treated with fPrP^WT^, Cu^2+^, Mn^2+^ and cation-bound fPrP^WT^. Figure S3: The determination of copper level of Cu-fPrP.

## References

[1] Soto C., Satani N. (2011). The intricate mechanisms of neurodegeneration in prion diseases. Trends Mol. Med..

[2] Gambetti P., Parchi P., Chen S.G. (2003). Hereditary Creutzfeldt-Jakob disease and fatal familial insomnia. Clin. Lab. Med..

[3] Will R.G. (2003). Acquired prion disease: Iatrogenic CJD, variant CJD, kuru. Br. Med. Bull..

[4] Prusiner S.B. (2012). Cell biology. A unifying role for prions in neurodegenerative diseases. Science.

[5] Wroe S.J., Pal S., Siddique D., Hyare H., Macfarlane R., Joiner S., Linehan J.M., Brandner S., Wadsworth J.D.F., Hewitt P. (2006). Clinical presentation and pre-mortem diagnosis of variant Creutzfeldt-Jakob disease associated with blood transfusion: A case report. Lancet.

[6] Llewelyn C.A., Hewitt P.E., Knight R.S.G., Amar K., Cousens S., Mackenzie J., Will R.G. (2004). Possible transmission of variant Creutzfeldt-Jakob disease by blood transfusion. Lancet.

[7] Prusiner S.B. (1998). Prions. Proc. Natl. Acad. Sci. USA.

[8] Bradford B.M., Mabbott N.A. (2012). Prion disease and the innate immune system. Viruses.

[9] Geschwind M.D. (2015). Prion Diseases. Continuum.

[10] Aguzzi A., Nuvolone M., Zhu C. (2013). The immunobiology of prion diseases. Nat. Rev. Immunol..

[11] Moore R.A., Herzog C., Errett J., Kocisko D.A., Arnold K.M., Hayes S.F., Priola S.A. (2006). Octapeptide repeat insertions increase the rate of protease-resistant prion protein formation. Protein Sci..

[12] Croes E.A., Theuns J., Houwing-Duistermaat J.J., Dermaut B., Sleegers K., Roks G., Van den Broeck M., van Harten B., van Swieten J.C., Cruts M. (2004). Octapeptide repeat insertions in the prion protein gene and early onset dementia. J. Neurol. Neurosurg. Psychiatry.

[13] Deloncle R., Fauconneau B., Guillard O., Delaval J., Lesage G., Pineau A. (2017). Copper brain protein protection against free radical-induced neuronal death: Survival ratio in SH-SY5Y neuroblastoma cell cultures. J. Trace Elem. Med. Biol..

[14] Yen C.F., Harischandra D.S., Kanthasamy A., Sivasankar S. (2016). Copper-induced structural conversion templates prion protein oligomerization and neurotoxicity. Sci. Adv..

[15] Hesketh S., Sassoon J., Knight R., Brown D.R. (2008). Elevated manganese levels in blood and CNS in human prion disease. Mol. Cell Neurosci..

[16] Johnson C.J., Gilbert P.U., Abrecht M., Baldwin K.L., Russell R.E., Pedersen J.A., Aiken J.M., McKenzie D. (2013). Low copper and high manganese levels in prion protein plaques. Viruses.

[17] Scheiber I.F., Mercer J.F.B., Dringen R. (2014). Metabolism and functions of copper in brain. Prog. Neurobiol..

[18] Singh N., Singh A., Das D., Mohan M.L. (2010). Redox control of prion and disease pathogenesis. Antioxid. Redox Signal..

[19] Salter M.W., Stevens B. (2017). Microglia emerge as central players in brain disease. Nat. Med..

[20] Davalos D., Grutzendler J., Yang G., Kim J.V., Zuo Y., Jung S., Littman D.R., Dustin M.L., Gan W.B. (2005). ATP mediates rapid microglial response to local brain injury in vivo. Nat. Neurosci..

[21] Cherry J.D., Olschowka J.A., O’Banion M.K. (2014). Neuroinflammation and M2 microglia: The good, the bad, and the inflamed. J. Neuroinflammation.

[22] Colton C., Wilcock D.M. (2010). Assessing activation states in microglia. Cns Neurol. Disord. Drug Targets.

[23] Colton C.A. (2009). Heterogeneity of microglial activation in the innate immune response in the brain. J. Neuroimmune Pharm..

[24] Le W., Rowe D., Xie W., Ortiz I., He Y., Appel S.H. (2001). Microglial activation and dopaminergic cell injury: An in vitro model relevant to Parkinson’s disease. J. Neurosci..

[25] Li R., Huang Y.G., Fang D., Le W.D. (2004). (-)-Epigallocatechin gallate inhibits lipopolysaccharide-induced microglial activation and protects against inflammation-mediated dopaminergic neuronal injury. J. Neurosci. Res..

[26] Sawada M., Suzumura A., Hosoya H., Marunouchi T., Nagatsu T. (1999). Interleukin-10 inhibits both production of cytokines and expression of cytokine receptors in microglia. J. Neurochem..

[27] Guo H., Callaway J.B., Ting J.P. (2015). Inflammasomes: Mechanism of action, role in disease, and therapeutics. Nat. Med..

[28] Schroder K., Tschopp J. (2010). The inflammasomes. Cell.

[29] Wang D., Duncan B., Li X., Shi J. (2020). The role of NLRP3 inflammasome in infection-related, immune-mediated and autoimmune skin diseases. J. Derm. Sci..

[30] Sarkar S., Rokad D., Malovic E., Luo J., Harischandra D.S., Jin H., Anantharam V., Huang X., Lewis M., Kanthasamy A. (2019). Manganese activates NLRP3 inflammasome signaling and propagates exosomal release of ASC in microglial cells. Sci. Signal..

[31] Yu K.H., Huang M.Y., Lee Y.R., Lin Y.K., Chen H.R., Lee C.I. (2020). The effect of octapeptid repeats on prion folding and misfolding.

[32] Bocharova O.V., Breydo L., Parfenov A.S., Salnikov V.V., Baskakov I.V. (2005). In vitro conversion of full-length mammalian prion protein produces amyloid form with physical properties of PrP(Sc). J. Mol. Biol..

[33] Lin S.J., Yu K.H., Wu J.R., Lee C.F., Jheng C.P., Chen H.R., Lee C.I. (2013). Liberation of GPI-anchored prion from phospholipids accelerates amyloidogenic conversion. Int. J. Mol. Sci..

[34] Choi C.J., Anantharam V., Saetveit N.J., Houk R.S., Kanthasamy A., Kanthasamy A.G. (2007). Normal cellular prion protein protects against manganese-induced oxidative stress and apoptotic cell death. Toxicol. Sci..

[35] Minutoli L., Puzzolo D., Rinaldi M., Irrera N., Marini H., Arcoraci V., Bitto A., Crea G., Pisani A., Squadrito F. (2016). ROS-Mediated NLRP3 Inflammasome Activation in Brain, Heart, Kidney, and Testis Ischemia/Reperfusion Injury. Oxid. Med. Cell Longev..

[36] Brown D.R. (2004). Metallic prions. Biochem. Soc. Symp..

[37] Brazier M.W., Davies P., Player E., Marken F., Viles J.H., Brown D.R. (2008). Manganese binding to the prion protein. J. Biol. Chem..

[38] Peres T.V., Schettinger M.R.C., Chen P., Carvalho F., Avila D.S., Bowman A.B., Aschner M. (2016). Manganese-induced neurotoxicity: A review of its behavioral consequences and neuroprotective strategies. BMC Pharm. Toxicol..

[39] Singh N., Asthana A., Baksi S., Desai V., Haldar S., Hari S., Tripathi A.K. (2015). The prion-ZIP connection: From cousins to partners in iron uptake. Prion.

[40] Stahl J.L., Greger J.L., Cook M.E. (1989). Zinc, copper and iron utilisation by chicks fed various concentrations of zinc. Br. Poult. Sci..

[41] Watt N.T., Taylor D.R., Kerrigan T.L., Griffiths H.H., Rushworth J.V., Whitehouse I.J., Hooper N.M. (2012). Prion protein facilitates uptake of zinc into neuronal cells. Nat. Commun..

[42] Summersgill H., England H., Lopez-Castejon G., Lawrence C.B., Luheshi N.M., Pahle J., Mendes P., Brough D. (2014). Zinc depletion regulates the processing and secretion of IL-1β. Cell Death Dis..

[43] Muroi M., Tanamoto K. (2015). Zinc- and oxidative property-dependent degradation of pro-caspase-1 and NLRP3 by ziram in mouse macrophages. Toxicol. Lett..

[44] Hafner-Bratkovič I., Benčina M., Fitzgerald K.A., Golenbock D., Jerala R. (2012). NLRP3 inflammasome activation in macrophage cell lines by prion protein fibrils as the source of IL-1β and neuronal toxicity. Cell Mol. Life Sci..

[45] Stoll G., Jander S., Schroeter M. (2000). Cytokines in CNS disorders: Neurotoxicity versus neuroprotection. J. Neural. Transm. Suppl..

[46] Beringue V., Demoy M., Lasmézas C.I., Gouritin B., Weingarten C., Deslys J.P., Andreux J.P., Couvreur P., Dormont D. (2000). Role of spleen macrophages in the clearance of scrapie agent early in pathogenesis. J. Pathol..

