# Supplementary material for: The Effects of Divalent Cation-Chelated Prion Fibrils on the Immune Response of EOC 13.31 Microglia Cells
Source: Cells. 2020 Oct 13;9(10):2285. doi: 10.3390/cells9102285 (PMC7602007; doi:10.3390/cells9102285)
Supplement: Supplementary file 1 [file cells-09-02285-s001.pdf]

Supplementary Materials

(a) (b)

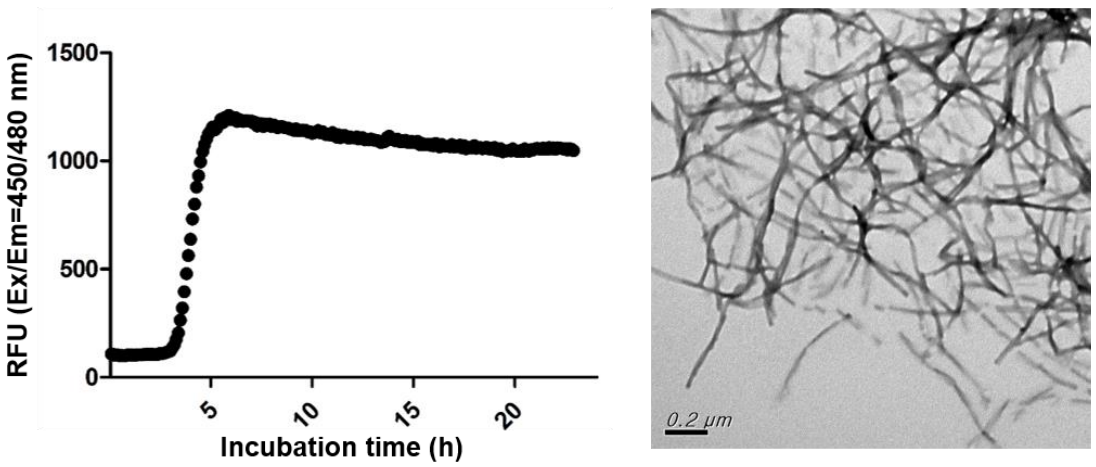

Figure S1. (a) The kinetics of fibril conversion monitored by fluorescence of ThT. (b) TEM images of the fibrils. Please find the details of the fibril conversion condition in materials and methods.

(a)

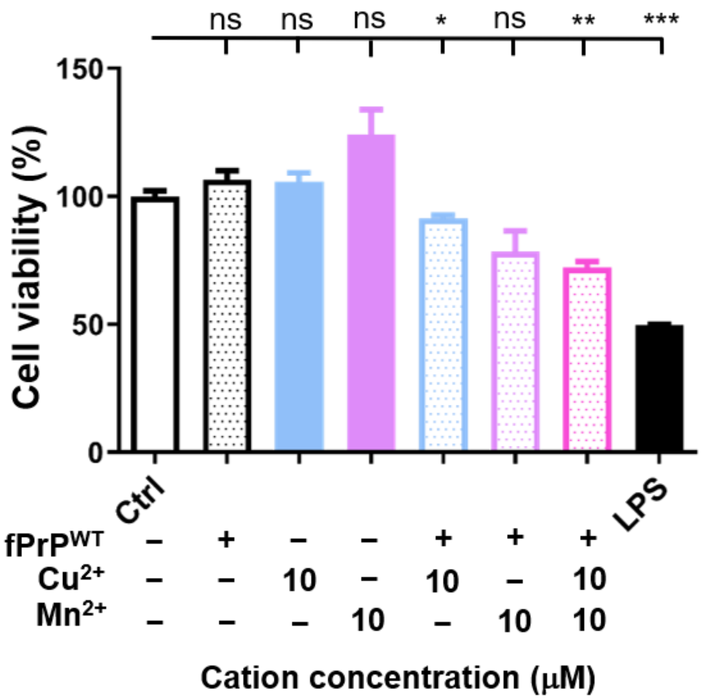

Figure S2. The cell viability of EOC 13.31 treat with $fPrP^{WT}$, Cu^{2+}, Mn^{2+} and cation-bound $fPrP^{WT}$.

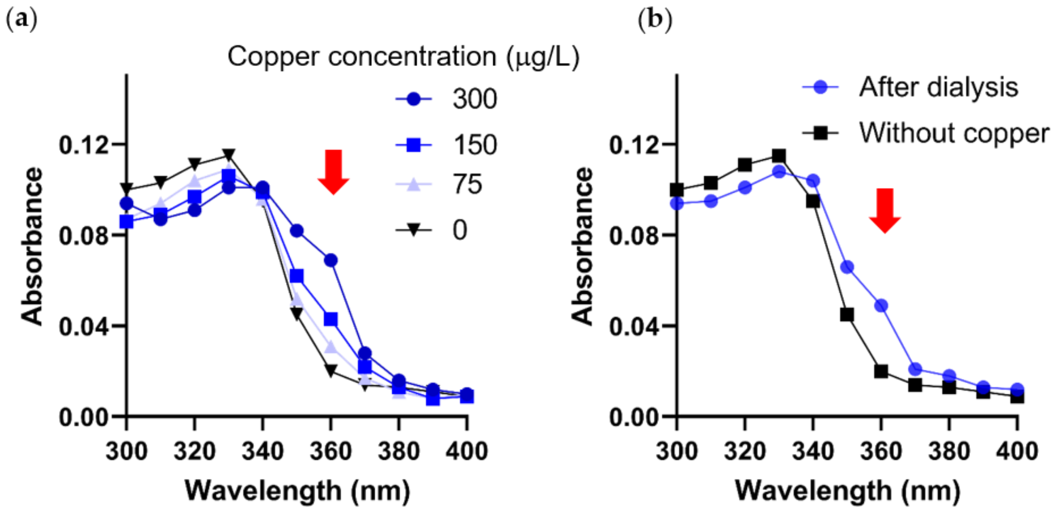

Figure S3. The determination of copper level of Cu-fPrP^{WT} by a Copper Assay Kit. (a) A test with different concentrations of Cu^{2+} standard solution. Copper chelation provides a significant absorption at 360 nm. (b) A test of Cu-fPrP^{WT}; free Cu^{2+} was removed from Cu-fPrP^{WT} sample by dialysis.
